# Distress-driven impulsivity interacts with trait compulsivity in association with problematic drinking: A two-sample study

**DOI:** 10.3389/fpsyt.2022.938275

**Published:** 2022-09-15

**Authors:** Chang Liu, Kristian Rotaru, Samuel R. Chamberlain, Murat Yücel, Jon E. Grant, Rico S. C. Lee, Teresa Wulandari, Chao Suo, Lucy Albertella

**Affiliations:** ^1^BrainPark, Turner Institute for Brain and Mental Health and School of Psychological Sciences, Monash University, Clayton, VIC, Australia; ^2^Monash Business School, Monash University, Caulfield, VIC, Australia; ^3^Department of Psychiatry, Faculty of Medicine, University of Southampton, Southampton, United Kingdom; ^4^Southern Health NHS Foundation Trust, Southampton, United Kingdom; ^5^Department of Psychiatry and Behavioral Neuroscience, University of Chicago, Chicago, IL, United States

**Keywords:** distress-driven impulsivity, negative urgency, compulsivity, alcohol use disorder, problematic drinking

## Abstract

**Objective:**

Problematic drinking is highly prevalent among the general population, oftentimes leading to significant negative consequences, including physical injury, psychological problems and financial hardship. In order to design targeted early interventions for problematic drinking, it is important to understand the mechanisms that render individuals at risk for and/or maintain this behavior. Two candidate drivers of problematic drinking are distress-driven impulsivity and trait compulsivity, with recent research suggesting these constructs may interact to enhance risk for addictive behaviors. The current study examined whether individual differences in distress-driven impulsivity and trait compulsivity interact in relation to problematic drinking.

**Method:**

Distress-driven impulsivity (indexed by the S-UPPS-P negative urgency subscale), trait compulsivity (indexed by the CHIT scale) and problematic drinking (indexed by the BATCAP alcohol scale) were assessed in two independent online samples (Sample 1, *n* = 117; Sample 2, *n* = 474). Bootstrapped moderation analysis was conducted to examine whether trait compulsivity moderated the relationship between distress-driven impulsivity and problematic drinking.

**Results:**

In both samples, there was a significant interaction between distress-driven impulsivity and trait compulsivity in relation to problematic drinking. Follow-up tests revealed that, in both samples, higher distress-driven impulsivity was associated with more problematic drinking behaviors among participants with high trait compulsivity only.

**Conclusions:**

The current findings add to the growing literature supporting an interactive relationship between impulsivity and compulsivity-related traits in relation to addictive behaviors and have implications for informing early detection of risk and targeted early interventions.

## Introduction

Problematic drinking [heavy and/or harmful drinking patterns (1)] is highly prevalent among the general population and may have a significant negative impact on individuals' physical and psychological health, as well as social and financial conditions (2). According to the World Health Organization report (3), alcohol use disorder causes three million deaths every year (accounting for 5.3% of all deaths) and contributes to the development and/or progression of more than 200 types of injuries and illnesses. For instance, a meta-analysis found that alcohol use disorders were strongly associated with major depression and anxiety disorders (4). Additionally, people experiencing problems with alcohol have significantly higher odds ratios for suicide attempts compared to those who did not consume alcohol (5). Other frequently reported issues resulting from problematic drinking include legal problems, impaired social functioning and financial hardship (2, 6). Given that problematic drinking is associated with a broad range of negative consequences, both personally and socially, it is important to understand the risk factors and underlying mechanisms that drive and maintain this behavior. This knowledge may help develop targeted prevention and early interventions for people experiencing problems with alcohol use.

Previous studies have suggested that certain personality traits, such as trait impulsivity [i.e., the tendency to act rashly without proper consideration of potential consequences (7)], may drive problematic drinking behaviors (8, 9). As a multi-faceted trait, impulsivity includes features such as the tendency to take rash action when distressed (distress-driven impulsivity), difficulties in remaining focused on a task (lack of preservation), the tendency to act without forethought (lack of premeditation), inability to stay focused on tasks (lack of perseverance), the tendency to seek novel, exciting experience (sensation seeking) and the tendency to act rashly under strong positive emotions (positive urgency). Among various facets of impulsivity, distress-driven impulsivity has been found to be consistently associated with alcohol use disorder and considered as a potential endophenotype for alcohol-related problems (8–10).

While impulsivity is a well-established risk factor for addictive behaviors and disorders (11), addictive behaviors and disorders can also be compulsive (12); that is, they feature repetitive behaviors that persist despite negative consequences (13, 14). Different accounts exist to explain compulsivity in addictive behaviors [e.g., (15–17)]. One line of thinking posits that there are individual differences in one's propensity to repetitive actions that persist despite negative consequences [e.g., trait compulsivity, (18)], which predispose individuals toward addictive behaviors. This idea is supported by studies showing that trait compulsivity is associated with a range of addictive behaviors, including problematic internet use and problematic drinking (19–21).

Despite distress-driven impulsivity and compulsivity both being associated with addictive behaviors, they are separable dimensional constructs and underlie distinct patterns of behavior (13, 22). Importantly, the proposed mechanisms through which distress-driven impulsivity and compulsivity influence addictive behaviors are different. For instance, individuals characterized by high distress-driven impulsivity may engage in a range of (potentially) addictive behaviors, with tendencies considered to reflect reduced control over impulses in the context of negative emotion (23, 24). On the other hand, individuals characterized by high trait compulsivity may repetitively engage in an addictive behavior despite that behavior being associated with adverse consequences, with such tendencies considered to reflect cognitive inflexibility and related processes (17, 25, 26).

An increasing number of studies suggest that distress-driven impulsivity- and compulsivity- related factors may interact to drive risk for problematic/addictive behaviors (24, 27). That is, while either factor alone might be related to engagement in addictive behaviors, when both co-exist at high levels, the addictive behaviors appear to be most problematic (24, 27). Importantly, findings of this interaction have led to explanations that offer mechanistic insights into the development of problematic addictive behaviors (27). Specifically, the interaction has been explained as reflecting inflexibility of a learned coping strategy. Briefly, as individuals high in distress-driven impulsivity are more likely to engage in problematic behaviors in the context of negative emotions [due to impaired cognitive control (28)], they are more likely to experience the stress-reducing consequences of the behavior (29, 30), which in turn can promote learning (31, 32) of that response as a coping strategy for the individual. Over time, engaging in addictive behaviors to cope with negative affect can lead to negative consequences. At this point, individuals high on trait compulsivity might be more likely to persist in engaging in these behaviors despite negative consequences, owing to inflexibility. In contrast, individuals with low trait compulsivity may find it easier to adjust their behavior in response to the changed circumstances (i.e., the onset of negative consequences) and engage in alternative coping strategies, owing to their greater flexibility.

To date, findings of this interaction have involved cognitive tasks to examine the moderating influence of inflexibility [a central component of compulsivity; (24, 27)]. Although cognitive tasks can be informative with respect to neurocognitive mechanisms, they can be time-consuming and difficult to administer. For these findings to be translated into practice, translatable, it would be ideal to replicate the interaction that has been shown to exist at a cognitive level, at a self-report level. Thus, to advance the understanding of mechanisms driving the persistence of problematic behaviors and determine whether the previously found interaction at the cognitive level (24) may be extended to the trait (self-report) level, the current study examined whether trait compulsivity interacts with (i.e., moderates) the relationship between distress-driven impulsivity and problematic drinking. We hypothesized that trait compulsivity will moderate the relationship between distress-driven impulsivity and problematic drinking. Specifically, in line with past research (24), we expect that distress-driven impulsivity will be related to problematic drinking among individuals with high trait compulsivity, whereas no such relationship will be seen among individuals with low trait compulsivity. Finally, to examine the robustness of this effect, the current study adopted a two-sample design. We examined the interaction between trait compulsivity and distress-driven impulsivity in two independent samples (i.e., a general community sample and a student sample).

## Method

Sample 1 consisted of 117 participants from the general community. Participants were recruited *via* the Mechanical Turk crowdsourcing platform. A brief description, including research purpose and survey design, was provided in the recruitment advertisement. Participants were included in the study if they were 18 years of age or older and provided informed consent. Eligible participants proceeded to an online survey. Participants who completed the survey received a USD $9 compensation.

Sample 2 consisted of 474 students. Participants were recruited through a student research participation pool at Monash Business School, Monash University, Australia. Course credit compensation was given for completing the study. Participants were included in the study if they were 18 years of age or older and provided informed consent.

For both study samples, the recruitment protocol followed the Declaration of Helsinki and was approved by the Monash University Human Research Ethics Committee. Descriptive statistics of study variables are presented in Table 1.

**Table 1:** Descriptive statistics.

	**Sample 1** ***N* = 117**	**Sample 2** ***N* = 474**
Age (mean, SD)	32.70 (6.78)	21.58 (2.51)
Female (*n*, %)	45 (38.5)	276 (58.2)
Distress-driven impulsivity (mean, SD)	7.98 (2.70)	9.59 (2.28)
Trait compulsivity (mean, SD)	37.79 (6.36)	31.88 (8.66)
Problematic Drinking (mean, SD)	3.88 (3.96)	2.39 (3.72)

### Measures

#### The Cambridge-Chicago compulsivity trait scale

This is a 15-item self-report scale assessing trait compulsivity. The CHIT scale covers reward drive, perfectionism and, most pertinent to the current study, inflexibility/rigidity (33). According to a recent review on measurements of impulsivity and compulsivity (34), the CHIT was recommended as a self-report compulsivity measure for future research. Participants were asked to rate how much they agree with the description from the scale ranging from strongly disagree (0) to agree /strongly agree (3). CHIT total score was the moderator of interest. Higher scores indicate higher levels of trait compulsivity.

#### The short version of the Urgency, premeditation (lack of), perseverance (lack of), sensation seeking, and Positive Urgency impulsivity behavior scale

This is a 20-item self-report scale assessing trait impulsivity. Subscales include Negative Urgency (4 items); Positive Urgency (4 items); Lack of Perseverance (4 items); Lack of Premeditation (4 items); and Sensation Seeking (4 items). Sample items for each subscale include “When I feel bad, I will often do things I later regret in order to make myself feel better now,” “I tend to lose control when I am in a great mood,” “Once I get going on something I hate to stop,” “I like to stop and think things over before I do them” and “I quite enjoy taking risks.” Participants were asked to rate how much they agree with the description from the scale ranging from agree strongly (1) to disagree strongly (4). Distress-driven impulsivity (Negative Urgency) subscale score was the independent variable of interest. The Negative Urgency subscale reflects the tendency to act rashly under negative emotions (35) and has been found to correlate with deficits in both general inhibitory control and specific response inhibition to negative emotional stimuli (28, 36). Higher scores indicate higher levels of distress-driven impulsivity.

#### Brief assessment tool of compulsivity associated problems

Problematic drinking was measured by the BATCAP alcohol use scale. This is a 6-item self-report scale assessing compulsivity-related problems associated with a given behavior (in this study, alcohol use). Individuals who reported having consumed alcohol in the past month were asked to complete the BATCAP for alcohol use. The BATCAP was developed to assess the severity of problems associated with any specified potentially compulsive or problematic behavior (34, 37). The six items in the BATCAP cover time lost, distress, loss of control, functional impact, anxiety if prevented from doing the behavior, and strongest urges in the past week. Participants were asked to rate how much they agree with the description on a Likert scale ranging from 0 to 4. The BATCAP alcohol total score was used to index problematic drinking. Higher scores indicate higher levels of problematic drinking. The BATCAP scale has been shown to be positively correlated to the Alcohol Use Disorders Identification Test [*rs* = 0.53, *p* < 0.01, (37)], indicating sufficient concurrent validity.

### Data analysis

To examine the interactive effect of distress-driven impulsivity and trait compulsivity on problematic drinking, as well as determine the moderating role of trait compulsivity in distress-driven impulsivity-alcohol use association, moderation analysis was conducted *via* PROCESS (38) in both samples. The PROCESS macro used a bias-corrected bootstrap method, which was suitable for analyzing non-normal distributed data (38). Negative urgency subscale score was entered as the independent variable, CHIT score was entered as the moderator, BATCAP alcohol score was entered as the dependent variable. Age, gender, and the other four S-UPPS-P subscales scores were set as covariates due to their potential confounding effect (37). Following the recommendations by Hayes (38), all continuous variables included in the analysis were mean centered. We applied 5,000 bootstrap samples for the current analysis. A significant interaction effect was identified when the 95% confidence interval did not contain zero. Simple slope tests were used to plot the interaction effect. The interaction was plotted at two levels of CHIT scores (high CHIT group: scored 1 SD above mean; low CHIT group: scored 1 SD below mean). The Johnson-Neyman regions of significance is included in the Supplementary material.

## Results

Sample 1 included 117 participants (38.5% female, *n* = 45), aged 18–50 (*mean* = 32.68, *SD* = 6.74). Sample 2 included 474 participants (58.2% female, *n* = 276), aged 18–44 years (*mean* = 21.58, *SD* = 2.51). Spearman's correlations across study variables are presented in Table 2. The results for moderation analysis are presented in Table 3. In Sample 1, we found a significant interaction effect between distress-driven impulsivity and trait compulsivity in predicting BATCAP alcohol score (*b* = 0.05, Boot *SE* = 0.02, *CI* = [<0.01, 0.08]). A similar interaction effect was also observed in Sample 2 (*b* = 0.02, Boot *SE* = 0.01, *CI* = [<0.01, 0.03]).

**Table 2:** Spearman's correlation across study variables.

**Variable**	**Mean**	**SD**	**1**	**2**	**3**	**4**	**5**	**6**	**7**
**Sample 1**
1. Distress-driven impulsivity	7.98	2.70							
2. Trait compulsivity	37.79	6.36	0.16**						
3. Problematic drinking	3.88	3.96	0.15**	0.10*					
4. Lack of premeditation	7.59	2.43	0.06	−0.18**	0.07				
5. Lack of perseverance	8.16	2.25	−0.11*	−0.36**	0.01	0.43**			
6. Positive urgency	6.96	2.74	0.56**	0.08	0.13**	0.17**	−0.03		
7. Sensation seeking	8.65	2.83	0.11*	0.16**	0.15**	−0.08	−0.14**	0.27**	
**Sample 2**
1. Distress-driven impulsivity	9.59	2.28							
2. Trait compulsivity	31.88	8.66	0.48**						
3. Problematic drinking	2.39	3.72	0.23*	0.16					
4. Lack of premeditation	8.07	1.89	0.36**	−0.04	0.12				
5. Lack of perseverance	8.27	1.79	0.06	−0.36**	0.15	0.38**			
6. Positive urgency	8.80	2.41	0.72**	0.34**	0.20**	0.50**	0.07		
7. Sensation seeking	10.43	2.30	0.37**	0.12	0.06	0.30**	0.07	0.41**	

Distress-driven impulsivity (measured using the S-UPPS-P negative urgency subscale); Trait Compulsivity (measured using the CHIT scale); Problematic Drinking (measured using the BATCAP Alcohol Use scale); Lack of Premeditation (measured using the S-UPPS-P lack of premeditation subscale); Lack of Perseverance (measured using the S-UPPS-P lack of perseverance subscale); Positive Urgency (measured using the S-UPPS-P positive urgency subscale); Sensation Seeking (measured using the S-UPPS-P sensation seeking subscale); ** signifies p < 0.01. *Signifies p < 0.05.

**Table 3:** Results of bootstrapped moderation analysis.

**Variable**	**B**	**BootSE**	**LLCI**	**ULCI**
**Sample 1**
Distress-driven impulsivity	0.30	0.24	−0.17	0.74
Trait compulsivity	0.14	0.07	<0.01	0.29
Interaction term	0.05	0.02	<0.01	0.08
Lack of premeditation	0.25	0.20	−0.13	0.65
Lack of perseverance	0.20	0.21	−0.15	0.67
Positive urgency	−0.03	0.24	−0.52	0.43
Sensation seeking	−0.04	0.11	−0.25	0.17
Age	0.09	0.05	−0.02	0.19
Gender	−0.79	0.68	−2.16	0.50
**Sample 2**
Distress-driven impulsivity	0.22	0.09	0.06	0.40
Trait compulsivity	0.03	0.02	−0.01	0.07
Interaction term	0.02	<0.01	<0.01	0.04
Lack of premeditation	0.10	0.09	−0.07	0.27
Lack of perseverance	0.18	0.11	−0.05	0.40
Positive urgency	0.21	0.09	0.03	0.39
Sensation seeking	0.13	0.07	−0.01	0.27
Age	0.18	0.11	<0.01	43
Gender	−0.98	0.40	−1.76	−0.22

Boot SE, Bootstrapped standard errors; LLCI and ULCI, 95% bootstrapped confidence intervals.

**Figure 1:**
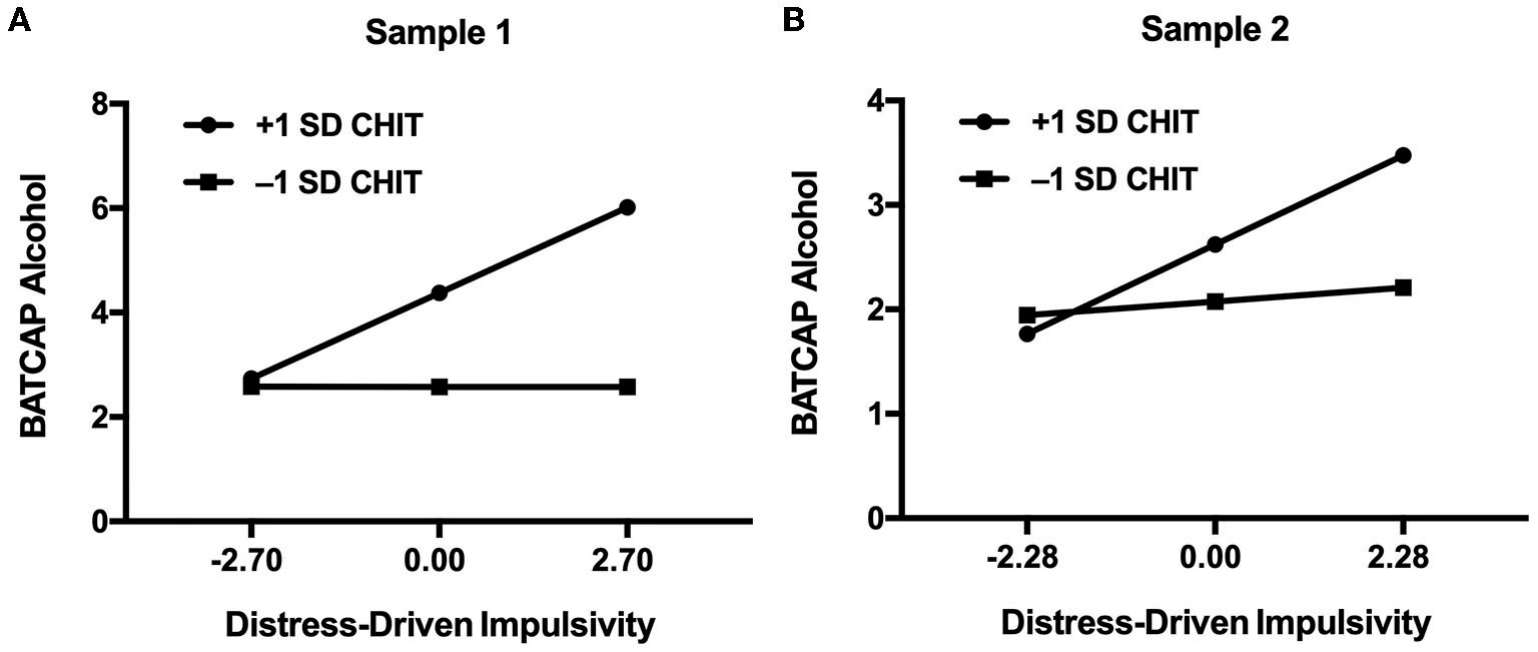
Effect of distress-driven impulsivity on problematic drinking at different levels of trait compulsivity in Sample 1 **(A)** and Sample 2 **(B)**.

Follow-up analysis in Sample 1 showed that the effect of distress-driven impulsivity the BATCAP alcohol score was significant in the high CHIT group only (*b* = 0.61, *SE* = 0.21, *CI* = [0.20, 1.02]). Similar results were observed in Sample 2 (*b* = 0.38, *SE* = 0.11, *CI* = [0.16, 0.60]). Specifically, in both samples, higher distress-driven impulsivity traits were associated with elevated problematic drinking among participants with high trait compulsivity only (Figure 1). No such association was found among participants with low trait compulsivity in both samples.

## Discussion

The current study aimed to examine whether there is an interaction effect between distress-driven impulsivity and compulsivity on problematic drinking and whether this effect could be replicated across two independent samples. Consistent with our hypothesis, we found that trait compulsivity moderated the association between distress-driven impulsivity and problematic drinking. Further, this effect was seen in both samples. Specifically, distress-driven impulsivity traits were associated with elevated problematic drinking among participants with high trait compulsivity only. No such association was found among participants with low trait compulsivity in both samples.

Impulsivity and compulsivity have long been viewed as risk drivers for problematic behaviors (11, 12, 18, 39). Growing evidence supports an interactive effect between impulsivity- and compulsivity-related cognition in driving addictive behaviors [e.g., (24)]. The current study extends previous research by showing that the interaction exists at the trait level. In line with previous interpretations [e.g., in relation to addiction-like eating; (27)], the current findings may be interpreted as: individuals with high distress-driven impulsivity levels are more prone to engage in problematic behaviors (e.g., alcohol consumption) under negative emotional states due to their deficits in cognitive control. Constantly engaging in drinking when distressed may increase individuals' likelihood of experiencing the stress-relieving effect of this behavior, leading individuals to acquire alcohol consumption as a coping strategy. Over time, such coping-motivated drinking may become maladaptive and lead to negative consequences. In view of negative consequences associated with drinking, individuals with low trait compulsivity may adjust their behaviors and engage in alterative coping strategies. Meanwhile, individuals with high trait compulsivity persist with the maladaptive option (i.e., alcohol consumption) due to impaired flexibility.

The current findings have important implications for the early identification of individuals at risk for problematic drinking. Specifically, by replicating the high-risk neurocognitive profile using self-report trait measures, the current findings highlight the potential of using a 5-min self-report trait screener to detect risk (a) simply (i.e., using measures that are easier to administer and less time-consuming than most cognitive tests) and (b) independently of drinking behaviors. This latter point has critical implications for early detection. Owing to the focus on traits as criteria to identify risk, such measures could be used to detect risk before drinking has even begun. In terms of developing targeted interventions, both impulsivity and compulsivity have been linked to impaired cognitive functioning (20, 24). Existing studies show that cognitive training provided promising outcomes for substance use disorders both in function improvement and symptom reduction (40, 41). Specifically, cognitive control training shows efficacy in reducing distress-driven impulsivity (42). Another potential type of intervention for reducing impulsivity and compulsivity could be personality change interventions. Digital personality change interventions (43) have been found effective in helping participants increase/decrease unwanted personality traits (indexed by the short Big Five Inventory-2) over 3 months, with medium- to large- effect sizes (43). Such interventions may be similarly effective in reducing trait impulsivity and compulsivity.

One key strength of the current study is utilizing a two-sample design. Specifically, the proposed relationship between distress-driven-impulsivity and trait compulsivity was examined in two different samples from two different countries with different demographics (e.g., general sample vs. student sample). The results were consistent in both samples, indicating the robustness of our findings. Despite its strength, several limitations should be noted when interpreting current findings. Firstly, the current study utilized cross-sectional samples, which restricted us from drawing any causal conclusions. Secondly, the generalizability of our results may be impacted due to the overall subclinical nature of our samples. Further, the utilization of the online crowdsourcing sample (i.e., the Mechanical Turk sample) with self-report measures may introduce potential issues of no verification of alcohol use compared to in-lab settings. Future studies should consider replicating our findings in clinical samples with structured clinical interviews. Finally, longitudinal studies are needed to examine the predictive effects of the impulsivity-compulsivity interaction.

In conclusion, the current study suggested that distress-driven impulsivity and trait compulsivity interactively determine the severity of problematic drinking. Trait compulsivity moderated the impulsivity-problematic drinking association. This is consistent with previous findings reporting the impulsivity-compulsivity interaction effect at the cognitive level was associated with increased problematic drinking behaviors. The current findings have important implications for future assessment and intervention efforts. Specifically, the short self-report measures used in the current study may be utilized to identify individuals at risk for problematic drinking, possibly even prior to the onset of symptoms. In turn, earlier identification of risk can help direct the appropriate support and early interventions to the people who would benefit most from them. Finally, the current findings highlight the potential of an emerging class of interventions (i.e., personality change interventions) for targeting trait impulsivity and compulsivity and, in turn, reducing risk for problematic drinking.

## Data availability statement

The raw data supporting the conclusions of this article will be made available by the authors, without undue reservation.

## Ethics statement

The studies involving human participants were reviewed and approved by Monash University Ethical Committee. The patients/participants provided their written informed consent to participate in this study.

## Author contributions

CL wrote the first draft of this manuscript. CL, KR, and LA designed the major components of the study. All authors contributed to the selection of study measures and revising subsequent versions of the paper.

## Funding

MY has received funding from Monash University, the National Health and Medical Research Council 302 (NHMRC; including Fellowship #APP1117188), the Australian Research Council (ARC), Australian Defense Science and Technology (DST), and the Department of Industry, Innovation and Science (DIIS). MY has also received philanthropic donations from the David Winston Turner Endowment Fund, Wilson Foundation. SC’s role in this study was funded by a Wellcome Trust Clinical Fellowship (110049/Z/15/Z & 110049/Z/15/A). RL was funded by a National Health and Medical Research Council project grant (APP1162031).

## Conflict of interest

Author MY has received payment from law firms in relation to court and/or expert witness reports. Author SC consults for Promentis and receives a stipend/honoraria from Elsevier for editorial work. Author JG has received research grants from Biohaven, Promentis, and Otsuka Pharmaceuticals. He also receives yearly compensation from Springer Publishing for acting as Editor-in-Chief of the Journal of Gambling Studies and has received royalties from Oxford University Press, American Psychiatric Publishing, Inc., Norton Press, and McGraw Hill. The remaining authors declare that the research was conducted in the absence of any commercial or financial relationships that could be construed as a potential conflict of interest.

## Publisher's note

All claims expressed in this article are solely those of the authors and do not necessarily represent those of their affiliated organizations, or those of the publisher, the editors and the reviewers. Any product that may be evaluated in this article, or claim that may be made by its manufacturer, is not guaranteed or endorsed by the publisher.
