# Supplementary material for: Distress-driven impulsivity interacts with trait compulsivity in association with problematic drinking: A two-sample study
Source: Front Psychiatry. 2022 Sep 15;13:938275. doi: 10.3389/fpsyt.2022.938275 (PMC9530652; doi:10.3389/fpsyt.2022.938275)
Supplement: Supplementary file 1 [file Table_1.docx]

**Table S1. Johnson-Neyman’s regions of significance**

| Sample 1 CHIT (centered) | Effect | SE | *t* | LLCI | ULCI |
| --- | --- | --- | --- | --- | --- |
| 15.21 | 1.03 | .30 | 3.43 | .43 | 1.63 |
| 13.41 | .94 | .28 | 3.41 | .40 | 1.49 |
| 11.61 | .86 | .25 | 3.37 | .35 | 1.36 |
| 9.81 | .77 | .23 | 3.29 | .31 | 1.24 |
| 8.01 | .69 | .22 | 3.14 | .25 | 1.12 |
| 6.21 | .60 | .21 | 2.92 | .19 | 1.01 |
| 4.41 | .51 | .20 | 2.60 | 1.22 | .91 |
| 15.21 | 1.03 | .30 | 3.43 | .43 | 1.63 |
| 13.41 | .94 | .28 | 3.41 | .40 | 1.49 |
| 11.61 | .86 | .25 | 3.37 | .35 | 1.36 |
| 9.81 | .77 | .23 | 3.29 | .31 | 1.24 |
| 8.01 | .69 | .22 | 3.14 | .25 | 1.12 |
| 6.21 | .60 | .21 | 2.92 | .19 | 1.01 |
| 4.41 | .51 | .20 | 2.60 | 1.22 | .91 |
| Sample 2 CHIT (centered) |  |  |  |  |  |
| 25.12 | .68 | .22 | 3.02 | .24 | 1.12 |
| 22.82 | .63 | .21 | 3.08 | .23 | 1.04 |
| 20.52 | .59 | .19 | 3.13 | .22 | .96 |
| 18.22 | .55 | .17 | 3.20 | .21 | .89 |
| 15.92 | .51 | .16 | 3.26 | .20 | .81 |
| 13.62 | .47 | .14 | 3.31 | .19 | .74 |
| 11.32 | .42 | .13 | 3.35 | .18 | .67 |
| 9.02 | .38 | .11 | 3.36 | .16 | .61 |
| 6.72 | .34 | .10 | 3.29 | .14 | .54 |
| 4.42 | .30 | .10 | 3.12 | .11 | .48 |
| 2.12 | .26 | .09 | 2.80 | .08 | .43 |

*Note.* *SE: Standard Errors; LLCI and ULCI: 95% confidence intervals*
